# Spinocerebellar ataxia type 11 (SCA11): *TTBK2* variants, functions and associated disease mechanisms

**DOI:** 10.1007/s12311-023-01540-6

**Published:** 2023-03-09

**Authors:** Daniela Felício, Mariana Santos

**Affiliations:** 1 UnIGENe, IBMC-Institute for Molecular and Cell Biology, i3S-Instituto de Investigação e Inovação em Saúde, Universidade do Porto, 4200-135 Porto, Portugal; 2 ICBAS, Instituto Ciências Biomédicas Abel Salazar, Universidade do Porto, 4050-313 Porto, Portugal

**Keywords:** Spinocerebellar ataxia type 11 (SCA11); Tau tubulin kinase 2 (TTBK2); Causal variant; Kinase activity; Cerebellar degeneration

## Abstract

Spinocerebellar ataxia type 11 (SCA11) is a rare type of autosomal dominant cerebellar ataxia, mainly characterized by progressive cerebellar ataxia, abnormal eye signs and dysarthria. SCA11 is caused by variants in *TTBK2*, which encodes tau tubulin kinase 2 (TTBK2) protein. Only a few families with SCA11 were described to date, all harbouring small deletions or insertions that result in frameshifts and truncated TTBK2 proteins. In addition, *TTBK2* missense variants were also reported but they were either benign or still needed functional validation to ascertain their pathogenic potential in SCA11. The mechanisms behind cerebellar neurodegeneration mediated by *TTBK2* pathogenic alleles are not clearly established. There is only one neuropathological report and a few functional studies in cell or animal models published to date. Moreover, it is still unclear whether the disease is caused by *TTBK2* haploinsufficiency of by a dominant negative effect of TTBK2 truncated forms on the normal allele. Some studies point to a lack of kinase activity and mislocalization of mutated TTBK2, while others reported a disruption of normal TTBK2 function caused by SCA11 alleles, particularly during ciliogenesis. Although TTBK2 has a proven function in cilia formation, the phenotype caused by heterozygous *TTBK2* truncating variants are not clearly typical of ciliopathies. Thus, other cellular mechanisms may explain the phenotype seen in SCA11. Neurotoxicity caused by impaired TTBK2 kinase activity against known neuronal targets, such as tau, TDP-43, neurotransmitter receptors or transporters, may contribute to neurodegeneration in SCA11.

**Supplementary Information:**

The online version contains supplementary material available at 10.1007/s12311-023-01540-6.

## Hereditary cerebellar ataxias

Hereditary cerebellar ataxias (HCAs) comprise a heterogeneous group of neurodegenerative disorders characterized by incoordination of movement, often associated with speech and eye movement disturbances. Symptoms are mainly caused by degeneration of cerebellar Purkinje neurons and spinocerebellar tracts. Additional neurological and non-neurological signs can also be present in patients [1, 2]. Currently, no effective pharmacological treatments are available for use in patients with HCAs, except for some episodic ataxias. The main problem in finding effective therapies is HCAs heterogeneity, which leads to a need for specific therapeutics focused on genotypes/disease mechanisms [2, 3]. Genetically, HCAs are very complex and can present with all modes of inheritance (mainly autosomal dominant and autosomal recessive, and a few forms of X-linked and mitochondrial inherited ataxias) [4, 5].

X-linked cerebellar ataxias (XLCAs) are a variable group of disorders mainly characterized by cerebellar dysgenesis (hypoplasia, atrophy, or dysplasia). XLCAs are caused by genomic imbalances or gene variants on the X chromosome, where more than 20 genes have been associated with cerebellar defects. These disorders are quite rare, except for fragile X syndrome, which is caused by CGG triplet expansions in the 5´UTR of *FMR1* [6].

Mitochondrial ataxias are clinically and genetically complex and can be caused by variants in mitochondrial DNA (mtDNA) or in nuclear genes that regulate mitochondrial function. Within the group of ataxias caused by mtDNA variants, there are multisystem disorders caused by point variants, namely myoclonic epilepsy and ragged red fibers (MERRF) and encephalomyopathy, lactic acidosis and stroke-like episodes (MELAS), or by large scale rearrangements, as in Kearns-Sayre syndrome (KSS) [7].

Autosomal recessive cerebellar ataxias (ARCAs) are a heterogeneous group of disorders caused by a multitude of genes [8]. Among more than 50 ARCAs, there are two forms caused by intronic repeat expansions that are relatively frequent: Friedreich’s ataxia caused by GAA repeat expansions in *FXN* [8] and cerebellar ataxia, neuropathy, vestibular areflexia syndrome (CANVAS) caused by a pentanucleotide repeat expansion in *RFC1* [9]. Other relatively common ARCAs, such as ataxia-telangiectasia (AT) and ataxia with oculomotor apraxia (AOA), and also rare forms are caused by conventional mutations [8]. Congenital ataxias and ataxias associated with metabolic disorders also have an autosomal recessive inheritance pattern [10].

Autosomal dominant cerebellar ataxias, also termed spinocerebellar ataxias (SCAs), are also a heterogeneous group of disorders with an intricate genotype–phenotype spectrum. Currently, these group include 50 SCA types, dentatorubral-pallidoluysian atrophy (DRPLA) and eight episodic ataxias (EA1-EA8). SCAs were numbered from SCA1 to SCA50 in the chronological order in which the disease *locus* was identified. However, only 44 *loci* are recognized since SCA9 and SCA33 are unassigned, SCA15, 16 and 29 were linked to the same *locus*, SCA19 and 22 were also connected to the same *locus* and SCA24 was proven to be recessively inherited (now referred as SCAR4), and considering *NPTX1* as the causal gene for SCA50 [11–13]. Moreover, there are five SCAs with known *loci* but with unknown causal genes (SCA4, 20, 30, 32, 39) [4, 14]. The genetic background of SCAs revealed different underlying mutational mechanisms: (1) coding repeat expansions of CAG triplets cause polyglutamine (polyQ) diseases (SCA1-3, 6, 7, 17 and DRLPA); (2) noncoding triplet, quintuplet or hexaplet repeat expansions (SCA10, 12, 31, 36 and 37) primarily cause disease at the RNA level; (3) other triplet coding repeat expansion can also generate a polyQ protein (SCA8, where a CTG expansion is transcribed in both directions); (4) conventional variants or rearrangements, such as missense variants (e.g., SCA13), frameshift variants (e.g., SCA11), deletions (e.g., SCA15) and duplications (e.g., SCA20), cause different SCAs forms [11, 14, 15]. One SCA-associated gene can be associated with more than one of these mechanisms, as is the case for *FGF14*. Missense and frameshift variants in *FGF14* have previously been reported to cause SCA27 but, very recently, an intronic GAA repeat expansion was found to interfere with *FGF14* transcription causing ATX-FGF14 [16, 17]. The polyQ ataxias are the most common SCAs. In most population-based analysis, SCA3/Machado-Joseph Disease is the most frequent subtype, but the relative frequency varies in different populations due to founder effects [18, 19].

Several cellular and molecular processes underlying HCAs pathogenesis have been identified, including transcriptional dysregulation, protein aggregation, autophagy impairment, alterations of calcium homeostasis and mitochondrial defects [8, 20]. However, the reason why all these mechanisms lead to cerebellar neurodegeneration remains largely elusive. Nevertheless, the identification of common pathogenic pathways between the different HCAs forms would be crucial for understanding the basis of cerebellar neurodegeneration [21, 22].

A more detailed description of HCAs clinical features, genetics and pathogenesis can found in Matilla-Dueñas et al*.* [4] and Klockgether et al*.* [15].

## Spinocerebellar ataxia type 11 (SCA11)

Spinocerebellar ataxia type 11 (SCA11) is a rare form of HCA with an autosomal dominant inheritance pattern. SCA11 prevalence is unknown, but it should account for less than 1% of SCAs in Europe [23]. SCA11 has been characterized as a slowly progressive cerebellar ataxia, with limb and gait imbalance, dysarthria, and oculomotor abnormalities. An overview of the main clinical findings in SCA11 cases is shown in Table 1. All affected individuals showed mild to severe cerebellar atrophy in both hemispheres and the vermis when examined by magnetic resonance imaging (MRI) [23–26]^.^ Positron emission tomography (PET) scan showed reduced metabolic activity in the cerebellum and pons in the Danish case [26]. In addition to cerebellar ataxia, SCA11 cases mostly presented dysarthria and ocular disturbance signs [23–26]. Pyramidal signs, such as hyperreflexia, were also observed in some patients [24–26]. The age of onset in SCA11 usually range from early to late adulthood, with an average of about 30 years (range from 4–64 years). The life span is thought to be normal, but most patients require walking aid (e.g., wheelchair) later in life [23, 26].

**Table 1:** Description of the SCA11 cases along with the identified *TTBK2* pathogenic truncating variants

Origin	*TTBK2* variant	Age of onset (Years old)	Main symptoms	Imaging findings	References
England	c.1329InsA p.Arg444fs*7	16–35	Imbalance; dysarthria; gait, truncal and limb ataxia; hyperreflexia; oculomotor disturbances	Cerebellar atrophy (both hemispheres)	[24]
Pakistan	c.1284_1285delAG p.Glu429fs*21	11–64	Imbalance; staggering; dysarthria; gait, truncal and limb ataxia; hyperreflexia; oculomotor disturbances	Cerebellar atrophy	
France	c.1306_1307delGA p.Asp435Tyrfs*14	35–58	Gait ataxia; dysarthria	Cerebellar atrophy (mainly affecting the vermis)	[23]
Germany	c.1306_1307delGA p.Asp436Tyrfs*14	40–50	Mild unsteadiness; gait ataxia; mild dysarthria; oculomotor disturbances	Pancerebellar atrophy	
England	c.1329InsA p.Arg444fs*7	17	No neurological features in addition to cerebellar ataxia	Cerebellar atrophy	[27]
Denmark	c.1205_1207delinsA p.Thr402Lysfs*48	4–9	Imbalance; gait ataxia; dysarthria; oculomotor disturbances; hyperreflexia	Cerebellar atrophy (both hemispheres and peduncles); olivo-pontine atrophy	[26]
Europe	c.1302dupC p.Arg435Glnfs*16	NA	Spastic ataxia	NA	[28]

The nucleotide and amino acid positions of *TTBK2* variants correspond to NM_173500.4. NA, not available

### SCA11-associated variants

So far, only a few families have been identified with SCA11. SCA11 was first described in a large British family as a slowly progressive pure cerebellar ataxia, genetically linked to the *locus* 15q14–21 [25]. Later, the *TTBK2* gene located at chromosome 15q15.2, which encodes tau tubulin kinase 2 (TTBK2) protein, was identified as the causal gene for SCA11 [24]. In this study, two heterozygous variants were identified in SCA11 patients: a one-base insertion in *TTBK2* (c.1329insA, p.Arg444Thrfs*7) in the British family and a deletion of a dinucleotide in *TTBK2* (c.1284_1285delAG, p.Glu429Aspfs*21) in another family of Pakistani ancestry [24]. Interestingly, in a 2013 next-generation sequencing (NGS) study, the variant present in the British family was identified in a patient with cerebellar atrophy, suggesting a possible link between them [27]. Three additional families with *TTBK2* truncating variants have been reported: one from France and another from Germany bearing the same variant (c.1306_1307delGA, p.Asp436Tyrfs*14) [23], and one from Denmark (c.1205_1207delinsA, p.Thr402Lysfs*48) [26]. Later, a different truncating variant (c.1302dupC, p.Arg435Glnfs*16) was identified by NGS in a patient of European origin with ataxia and spasticity signs [28]. A detailed description of the SCA11 cases along with the identified *TTBK2* pathogenic variants can be found in Table 1.

More recently, some heterozygous missense variants in *TTBK2* (Table 2) have been reported in patients presenting with cerebellar ataxia [28–36]. However, as far as we understand, none of them were clearly evaluated as pathogenic (pathogenicity predictions and conservations scores of these *TTBK2* missense variants are shown in Table S1). Indeed, two of these variants were reported as being normal rare variants [31], while several other variants were classified as variants of unknown significance [28, 33, 36]. Moreover, in any of these reports, functional studies were performed to prove the detrimental effect of the missense variants.

**Table 2:** Description of the inherited cerebellar ataxia cases with *TTBK2* missense variants identified (mostly variants of unknown significance)

Origin	*TTBK2* variant	Age of onset (Years old)	Main clinical findings	References
Afghanistan	c.2525A > G p.Glu842Gly (a, b)	9 – 40 s	Progressive gait ataxia; orthostatic dysregulation; nystagmus; neuropathy; ponto-cerebellar atrophy	[31]
Germany	c.3329G > A p.Arg1110His (b)	72	Gait ataxia; tremor; nystagmus; feet hyperkinesia; cerebellum atrophy; slight cortical atrophy	
Norway	c.245G > C p.Gly82Ala (c)	NA	Complex ataxia; spastic ataxia	[33]
Korea	c.3467G > A p.Arg1156Gln	53	Episodic ataxia; vertigo;	[35]
European	c.3658G > A p.Gly1220Ser (c)	NA	Ataxia (Patients with dominantly inherited cerebellar ataxias from the SPATAX network)	[36]
	c.3515G > T p.Gly1172Val (c)	NA		
	c.2980G > T p.Asp994Tyr (c)	NA		
	c.1173C > G p.Asn391Lys (c)	NA		
	c.659 T > C p.Val220Ala (c)	NA		
	c.2912A > G p.Lys971Arg (c)	NA		
European	c.3406A > G p.Asn1136Asp (c)	NA	Pure ataxia	[28]
Qatar	c.2030C > G p.Thr677Arg (d)	NA	Global developmental delay, mental retardation	[34]
	c.3526C > T p.His1176Tyr (d)	NA		
China	c.2831G > A p.Arg944Gln	11	Ataxia; slurred speech; dysgraphia; learning disability	[32]
China	c.3290 T > C p.Val1097Ala	38–44	Gait ataxia; dysarthria; dysphagia, nystagmus; muscle atrophy; moderate cerebellum and medulla oblongata atrophy	[29]
Italy	c.239 T > A p.Phe80Tyr	73	Spinocerebellar ataxia	[30]

^(a)^The index patient also had pathogenic expanded CAG repeats in *ATXN3* (SCA3/MJD)

^(b)^The authors considered not to be enough evidence to support variant pathogenicity

^(c)^The authors classified the variants as variants of unknown significance

^(d)^Compound heterozygous variants

The nucleotide and amino acid positions of *TTBK2* variants correspond to NM_173500.4. NA, not available. See table S1 for pathogenicity and conservation predictions and American College of Medical Genetics and Genomics (ACMG) classification of these missense variants

## Tau tubulin kinase 2 (TTBK2) protein

**Fig. 1:**
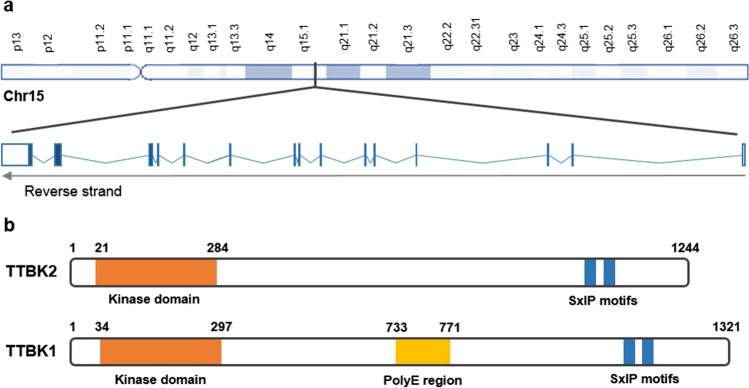
***TTBK2* genomic location and protein domains. (a)** *TTBK2* gene is located at chromosome 15q15.2 and has 14 coding exons (exon 1 is non-coding). **b** TTBK1 (amino acids 34–297) and TTBK2 (amino acids 21–284) have highly similar N-terminal kinase domains (88% identity and 96% similarity). At the C-terminal end, both TTBK1 and TTBK2 have serine-rich polypeptide regions containing SxIP (serine-any amino acid-isoleucine-proline) motifs. TTBK1 also has a central polyglutamate (polyE) region

TTBK2 is a serine-threonine protein kinase that belongs to the casein kinase (CK1) group of eukaryotic protein kinases [37]. The other TTBK family member is TTBK1, which is encoded by a different gene (*TTBK1*) and is neuron specific. The N-terminal kinase domains of TTBK1 and TTBK2 (Fig. 1) have 88% identity and 96% similarity. The rest of their sequence has only 35% identity and 63% similarity, but there is a small C-terminal domain with 43% identity and 58% similarity [38]. Both TTBK1 and TTBK2 have at least two serine any amino acid isoleucine proline (SxIP) motifs at their C-terminal region (Fig. 1b), which are recognized by end binding proteins (EB) proteins [39, 40]. Although TTBK1 and TTBK2 are conserved among vertebrates, only the catalytic domain is preserved in invertebrates, suggesting that *TTBK1* and *TTBK2* diversified from a common shorter *TTBK* gene during evolution [38].

*TTBK2* can encode multiple protein isoforms by alternative splicing; the longest has 1244 amino acids and an expected molecular weight of 137 kDa. TTBK2 protein is ubiquitously expressed in human adult and fetal tissues [24]. In particular, it is highly expressed and exhibit high kinase activity in brain and testis [41]. Within the brain, TTBK2 is expressed in all regions, with high expression in the Purkinje and granular cell layers of the cerebellum, the hippocampus, the midbrain, and the substantia nigra. On other hand, low expression levels were detected in the brain cortex [24].

**Fig. 2:**
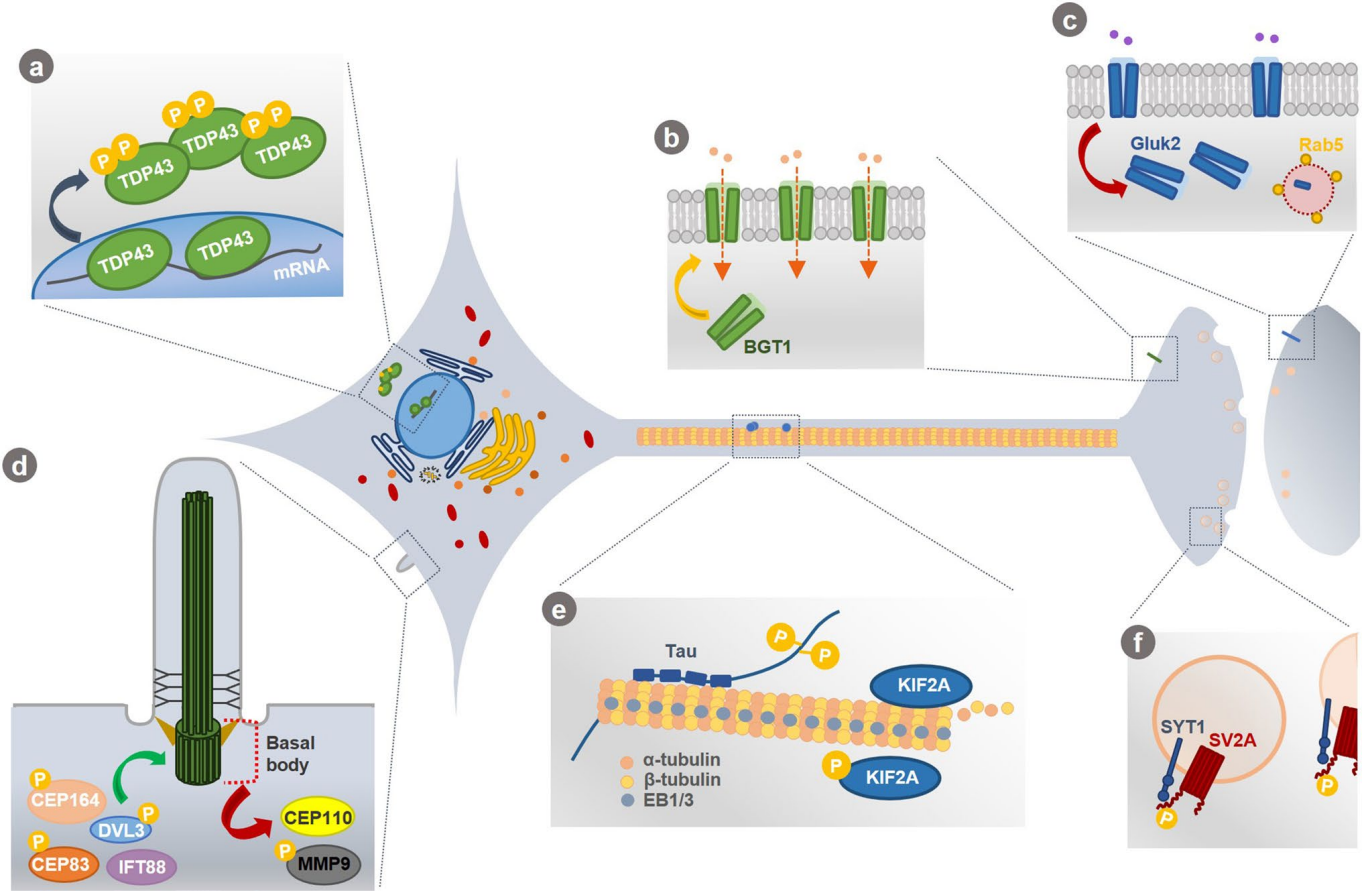
**TTBK2 targets and associated cellular processes. (a)** TDP-43, a DNA/RNA binding protein, continuously shuttles between the nucleus and cytoplasm. TTBK2 phosphorylates TDP-43, which can lead to TDP-43 recruitment into cytoplasmic inclusions. **(b)** Through an unknown mechanism, TTBK2 is able to increase BGT1 abundance at the plasma membrane, enhancing GABA-induced inward current. **(c)** Conversely, TTBK2 seems to stimulate Gluk2 internalization through Rab5-dependent endocytosis, decreasing Gluk2 protein abundance at the plasma membrane and the glutamate-induced currents. **(d)** TTBK2 is critical for ciliogenesis initiation from the basal body, namely by phosphorylating and/or recruiting several essential proteins in the process (e.g., CEP164, CEP83, DVL2/3, IFT88) and by promoting CP110 removal from the mother centrioles. Also, MPP9 phosphorylation by TTBK2 promotes its own proteasomal degradation and further removal of the CP110-CEP97 complex, promoting ciliogenesis. **(e)** TTBK2 participates in microtubules dynamics regulation by phosphorylating β-tubulin, tau and KIF2A. TTBK2 also binds to EB1/3 that allows KIF2A phosphorylation, which in turn inhibits KIF2A/microtubules interaction and decreases KIF2A microtubule depolymerizing activity. **(f)** TTBK2 phosphorylates SV2A within cluster 2, thus promoting SV2A binding SYT1, which is essential for SYT1 retrieval during synaptic vesicle endocytosis

TTBK2 was first identified based on its ability to phosphorylate the microtubule-associated proteins tau and β-tubulin in vitro [42, 43]. Since then, several cellular functions have been attributed to TTBK2, but few have been described in detail. TTBK2 potential functions include: (1) ciliogenesis [44]; (2) regulation of microtubule dynamics [40]; (3) modulation of membrane transporters and receptors [45–47]; (4) phosphorylation of the transactive response DNA-binding protein 43 kDa (TDP-43) [48]; (5) mitosis [49, 50]; (6) cancer progression [51]; (7) and maintenance of the connectivity and viability of Purkinje cells [52]. A summarised description of TTBK2 targets in these cellular processes can be found in Fig. 2.

### TTBK2 role in microtubules dynamics

Microtubules are dynamic structures composed of α/β tubulin heterodimers tightly controlled to ensure the normal function and division of eukaryotic cells. In addition to the phosphorylation of β-tubulin and tau protein [42, 43], TTBK2 also acts as a plus-end tracking protein (+ TIP) by tracking growing microtubules ends in a kinase-independent manner [39]. TTBK2 binds to the end binding proteins 1/3 (EB1/3), which enables the phosphorylation of kinesin family member 2A (KIF2A) (Fig. 2e) at Ser135 in vitro, inhibiting KIF2A interaction with the microtubules and decreasing KIF2A microtubule depolymerizing activity [40]. In the absence of TTBK2, KIF2A exhibit an extensive localization to the microtubules, which induces short-lived microtubules with an increased shrink rate and a decreased rescue frequency. On the other hand, overexpression of TTBK2 displaces EB1 from the microtubules, suggesting that it may regulate the association of + TIPs and other microtubule-associated proteins with newly polymerized ends. Therefore, the EB–TTBK2 interaction appears to antagonize the microtubule-depolymerizing machinery, primarily affecting KIF2A [40].

One study also suggested that centrosomal protein of 164 kDa (CEP164) may play a role in recruiting TTBK2 to the midbody during cytokinesis [53]. Although, neither this theory was confirmed nor the role of TTBK2 at the midbody [50, 53]. Another study reported the TTBK homolog in *D. melanogaster* (Asator) to be localized in the cytoplasm during interphase but redistributed to the mitotic spindle during mitosis, where it interacts with the spindle matrix protein Megator (homolog of the human translocated promotor region protein) that take part in the spindle assembly checkpoint. Thus, it was proposed that Asator kinase activity may regulate spindle function and microtubule dynamics [49].

### TTBK2 function in ciliogenesis

TTBK2 plays a pivotal role in the initiation of ciliogenesis from the basal body (Fig. 2d). TTBK2 is localized at the distal end of the mother centriole, where it is responsible for the removal of the centriolar coiled coil protein 110 (CP110; negative regulator) and recruitment of the intraflagellar transport 88, 140 and 81 proteins (IFT88, IFT140 and IFT81; positive regulators and cargo carriers in the cilium) [44, 54, 55]. It has also been suggested that TTBK2 regulates a final step in cilia initiation by recruiting Inturned (INTU; core subunit of the ciliogenesis and planar polarity effector complex) to the mother centriole, which in turn recruits the small guanosine triphosphatase RSG1 (positive regulator) [56]. Indeed, TTBK2 localization at the mother centriole is a critical step in ciliogenesis initiation, being mediated by CEP164 [55, 57].

In addition to the basal body, TTBK2 is also found at the transition zone of the cilium, where it could regulate the maintenance of ciliogenesis [44, 54]. Indeed, Bowie et al*.* reported that TTBK2 also plays a role in controlling cilia length, stability, and trafficking [54]. They showed that TTBK2 hypomorphic mutant cells present decreased cilia length and frequency of cilia formation, and disrupted trafficking of the Sonic Hedgehog (SHH) downstream effector Smoothened (SMO) and regulator KIF7 (controls cilium structure) [54].

Furthermore, ciliogenesis significantly depends on TTBK2 kinase activity [44]. TTBK2 phosphorylates several proteins required for ciliogenesis initiation, such as CEP164, CEP83, CEP89, Rabin8, coiled-coil domain containing 92 (CCDC92) and dishevelled 2/3 (DVL2/3), in their intrinsically disordered regions (IDRs) [58]. Although the effect of TTBK2-dependent phosphorylation on these proteins was not determined, it was hypothesized that it may regulate protein interactions and complexes necessary for primary cilium formation [58]. In accordance with this, *Oda *et al*.* have shown that CEP164 and/or DVL3 phosphorylation by TTBK2 inhibited CEP164-DVL3 interaction [57]. Moreover, Lo et al*.* reported that CEP83 phosphorylation by TTBK2 controls membrane vesicle docking, which promotes CP110 removal from the mother centrioles and further ciliogenesis initiation [59]. Other interesting effect of TTBK2-dependent phosphorylation may be targeting ciliary proteins for degradation [60]. Phosphorylation of M-Phase Phosphoprotein 9 (MPP9) by TTBK2 at the beginning of ciliogenesis promotes proteasomal degradation of MPP9 (a negative regulator of ciliogenesis). This facilitates the removal of MPP9 and the CP110-CEP97 complex (negative regulator) from the distal end of the mother centriole, promoting the initiation of ciliogenesis [60].

Therefore, TTBK2 appears to play a major role in controlling cilia initiation, maintenance, stability, and trafficking through phosphorylation and/or protein interactions.

### TTBK2 role in neuronal processes

One of the main targets of TTBK2 is the cytoskeletal tau protein [43]; abnormal tau hyperphosphorylation is associated with neurofibrillary tangles (NFTs) formation and neurodegeneration in Alzheimer’s Disease [61]. Interestingly, the two TTBK2-dependent phosphorylation sites in tau (Ser208 and Ser210) have been reported as elevated phosphorylation sites in Alzheimer’s disease brain [62, 63]. Moreover, tau phosphorylation at Ser208 increased tau affinity towards microtubules and contributed to tau aggregation, being a unique marker of aggregation and mature NFTs compared to other phosphorylation sites [63]. Intracellular aggregates of hyperphosphorylated tau are also a hallmark of frontotemporal lobar degeneration associated with tau inclusions (FTLD-tau). Elevated levels of TTBK2 and increased immunoreactivity of TTBK2 in the frontal cortex and hippocampus were observed in post-mortem tissues from FTLD-tau patients [64]. Evidence of tau-related neurodegeneration promoted by TTBK2 was observed in *C. elegans* models. One study reported that knocking down the TTBK2 *C. elegans* homolog (TTBK) in the presence of a mutant tau transgene (linked to FTLD and parkinsonism linked to chromosome 17) enhanced worms uncoordinated movement phenotype [65]. A second study showed that co-expression of human TTBK2 in a human tau background in *C. elegans* reduced worms lifespan, exacerbated behavioural defects and cause a significant loss of GABAergic motor neurons and axonal abnormalities [64]. Moreover, high levels of TTBK2 were lethal in combination with high levels of tau in worms. In the same study, other potential TTBK2-dependent phosphorylation sites in tau were identified (Thr181, Ser202, Thr231, and Ser396/404) [64].

TTBK2 has also been found to phosphorylate TDP-43, a DNA/RNA binding protein, at Ser409/410 in vitro and in vivo [48]. Loss of functional TDP-43 in the nucleus and accumulation of hyperphosphorylated and ubiquitinated TDP-43 in cytoplasmic inclusions are hallmarks of amyotrophic lateral sclerosis (ALS) and FTLD associated with TDP-43 inclusions (FTLD-TDP). TDP-43 positive inclusions have been also detected in patients with other neurological disorders [66]. Overexpression of TTBK1 and TTBK2 in cells can induce TDP-43 phosphorylation and recruitment into cytoplasmic inclusions (Fig. 2a), similar to the neuropathology seen in neurological disorders [48]. Moreover, TTBK2 immunostaining is increased in FTLD-TDP frontal cortex compared to controls, and TTBK2 co-localize with TDP-43 positive inclusions in FTLD-TDP frontal cortex and ALS spinal cord [48, 64].

Other neuronal targets of TTBK2 include the synaptic vesicle protein 2A (SV2A) [67], the betain/GABA transporter 1 (BGT1) [45] and the glutamate (kainate) receptor subunit 2 (GluK2) [47].

Both TTBK1 and TTBK2 can phosphorylate SV2A at two clusters of sites in the N-terminal cytoplasmic region but TTBK2 is more effective in phosphorylating the Thr84 residue within cluster 2 (Fig. 2f). This event is critical in promoting SV2A binding to the Ca^2+^ binding domain C2B of synaptogamin-1 (SYT1), a Ca^2+^ sensor that triggers neurotransmitter release. The interaction between SV2A and SYT1 is essential for the specific retrieval of synaptotagmin-1 during synaptic vesicle endocytosis [67].

TTBK2 seems to have opposite effects on BGT1 and GluK2 activities in *Xenopus* oocytes models. Co-expression of TTBK2 with BGT1 enhanced GABA induced currents [45] while co-expression with GluK2 decreased glutamate-induced currents [47]. Upregulation of BGT1 and downregulation of GluK2 by TTBK2 is mediated, at least in part, by respectively increasing or decreasing BGT1 and GluK2 protein abundances at the cell membrane (Fig. 2b and c) [45, 47]. Moreover, it is possible that TTBK2 stimulates Gluk2 internalization through small G-protein (GTPase) Rab5-dependent endocytosis, ultimately mediating neuroexcitotoxicity protection [47].

Therefore, it is possible that TTBK2 contributes to synapse homeostasis/stability through modulation of synaptic vesicles transport, and neuronal receptors and transporters localization and activity.

## SCA11 pathogenesis

Despite all the functions attributed to TTBK2, the mechanisms underlying cerebellar neurodegeneration in SCA11 are still not clearly stablished. Moreover, there is only one neuropathological report on SCA11. *Post-mortem* neuropathological examination of the brain of one affected individual from the British family (c.1329insA) revealed gross atrophy of the cerebellum accompanied by severe loss of Purkinje cells and granule cells, mild gliosis of the cerebellar white matter, and neuronal loss in the dentate nucleus [24, 68, 69]. The brainstem was well preserved, while the medulla revealed severe neuronal loss, and the cerebral hemispheres revealed atrophy consistent with the patient’s age (77 years old) [68, 69]. There were other alterations related with aging, such as, neurofibrillary tangles and β-amyloid-positive plaques within the hippocampus, neocortex, and transentorhinal, entorhinal and insular cortex. Neuronal intranuclear or cytoplasmic inclusions positive for p62 or ubiquitin were not detected [24, 68]. This neuropathological report is unique amongst SCA11 cases.

Functional studies showing the impact of *TTBK2* variants are scarce and include two with non-concordant results [24, 41]. Lymphoblasts from SCA11 patients (c.1329insA; c.1284_1285delAG) presented mRNA levels reduced by approximately 50%, when compared to unaffected individuals, suggesting that mutated mRNA is prematurely degraded by nonsense-mediated decay (NMD), causing TTBK2 haploinsufficiency. Still, it seems that a proportion of the abnormal mRNA escapes NMD [24]. Contrarily, at the protein level, overexpression studies showed that the same TTBK2 truncating mutants were expressed at higher levels compared to wild-type TTBK2. Moreover, TTBK2 mutants partially promoted the relocalization of TTBK2 from the cytosol to the nucleus and suppressed its kinase activity [41]. Additional studies showed that a truncated TTBK2 form (at residue 450) is kinase inactive, not being able to regulate the activities of a membrane receptor and a transporter in *Xenopus* models [45, 47].

Interestingly, homozygous mutant mice for the *TTBK2* truncating variant c.1329insA were embryonic lethal after embryonic day 10. Embryos at day 10 showed major abnormalities, such as developmental delay, indistinct brain subdivisions and distorted caudal bodies. On the other hand, heterozygous mutant mice for the same variant were viable and fertile, with no apparent abnormalities, and a regular lifespan [41]. Moreover, analysis of TTBK2 kinase activity showed a 40–50% reduction in the brain of heterozygous mutant mice and nearly a 90% reduction in mouse embryonic fibroblasts (MEFs) from homozygous mutant mice [41].

Additionally, some studies have suggested that SCA11 truncating variants cause abnormalities in ciliogenesis [44, 54, 55], which is possibly the most well studied function of TTBK2 (described above). Primary cilia are microtubule-based extensions of the plasma membrane that are linked to hereditary developmental syndromes termed ciliopathies [70]. Primary cilia formation is triggered at the distal end of the mother centriole (basal body) during the quiescent state (G0 phase) [71]. In MEFs derived from mice bearing a *TTBK2* null mutation, ciliogenesis is blocked because TTBK2 is required for ciliogenesis initiation by removing the negative regulator CP110 from the mother centriole and by recruiting IFT proteins [44]. In the same way, heterozygous and homozygous embryos mice for a SCA11 truncated variant (c.1329insA) lacked cilia in most tissues [54]. Rescue experiments showed that while wild-type TTBK2 overexpression promoted cilia formation in *TTBK2* null MEFs, the same did not occur in homozygous mutant mice, in which the frequency of cilia rescue was significantly lower. Moreover, SCA11 truncated proteins (c.1329insA; c.1284_1285delAG) lacked activity and seemed to interfere with the function of wild-type TTBK2 in ciliogenesis, pointing to a dominant negative effect of TTBK2 truncated forms [44, 54]. Nevertheless, these truncated proteins were stable and partially retained a normal centrosomal localization [44]. Another study reported that CEP164 is responsible for recruiting TTBK2 to centrioles, thus promoting ciliogenesis. However, the SCA11-truncated protein (c.1329InsA) was unable to bind to CEP164, which might explain the partial localization defects of TTBK2 truncated proteins in cells [55].

In overall, these functional studies point to both a loss of function and a dominant negative effect of TTBK2 truncating variants but mostly focus on the role of TTBK2 during ciliogenesis.

## Conclusions

SCA11 is a rare form of HCA caused by heterozygous small deletions or insertions in *TTBK2* (Table 1), generating truncated proteins with intact N-terminal kinase domains. Nevertheless, loss of the C-terminal region may abolish other functional domains, yet uncharacterized, and/or lead to conformational changes that impact TTBK2 stability, interactions, and functions. Thus, SCA11 has been linked to both haploinsufficiency [24] and a dominant negative mechanism where mutated TTBK2 interferes with the normal allele functions [44]. Additionally, *TTBK2* missense variants were reported in patients with cerebellar ataxia but, in our understanding, additional studies are needed to validate their pathogenicity (Table 2 and S1). Validation of new variant types may uncover new disease mechanisms behind SCA11 and expand the number of SCA11 cases. The most well studied mechanism disrupted by *TTBK2* truncating variants is perhaps ciliogenesis [44, 54, 55], where TTBK2 plays a pivotal role, particularly in cilia formation. Pathogenic variants in ciliary proteins can result in early embryonic lethality or severe diseases termed ciliopathies [72]. Most of these disorders are inherited in an autosomal recessive manner with symptoms start during childhood or adolescence but some are more genetically complex, presenting digenic inheritance, modifiers genes or gene-dosage effects [72]. Since SCA11 and ciliopathies genetic inheritance and phenotypes seem to differ quite substantially, it is possible that other disease mechanisms underlie SCA11 in addition to ciliary defects [50]. The role of TTBK2 as + TIP and regulator of tubulin, tau or KIF2A through phosphorylation [40, 42, 43] also points to a disturbance in microtubule-based and axonal transport in SCA11. Subsequently, TTBK2 may play a role in synaptic vesicles transport and neurotransmitter release [67], and also in controlling the transport process and activity of neuronal receptors and transporters [45, 47]. Although, additional studies are needed to explore these functions and their implications in SCA11.

### Supplementary Information

Below is the link to the electronic supplementary material.

Supplementary file1 Table S1 Pathogenicity and conservation predictions of the TTBK2 missense variants reported in patients with inherited cerebellar ataxia.CADD, REVEL and DANN scores are considered as deleterious above 20, 0.5, and 0.5, respectively. In nucleotide conservation scores, the larger the score, the more conserved the site. GERP++ RS, PhastCons (17, 30 and 100way), PhyloP100way, Phylo30way,and Phylo17way range from -12,3 to 6,17; 0 to 1; -20 to 10,003; -20 to 1,312; and -13,362 to 0,756, respectively.MAF, minor allele frequency in Gnomad v2.1.1. NA, not available. Nº software, the number of software predicting a deleterious effect amongst all the prediction software for the variants. * PP4 criteria was attributed in all cases considering that all patients’ phenotypes are specific for SCA11; PP3 criteria was attributed to missense variants with Nº software > 7 (from 14 in total); BP4 criteria was attributed to missense variants with Nº software ≤ 7 (from 14 in total); PM1 criteria was considered for variants affecting TTBK2 kinase domain. ** The variant was considered of uncertain significance but borderline likely pathogenic. (XLSX 15 KB)

## Data Availability

Not applicable.
